# Unveiling the roles of SPP1^+^ macrophage and IGFBP2^+^ fibroblast in lung adenosquamous carcinoma through single-cell analysis

**DOI:** 10.1016/j.gendis.2025.101779

**Published:** 2025-07-24

**Authors:** Yao Lin, Yaxin Chen, Dandan Xiong, Jing Huang, Hongmo Liu, Yawen Qi, Jinfeng Chen, Jun Meng, Yueqi Li, Jingyuan Yang, Yi Bao, Wenxing Li, Li Yang, Sanqi An

**Affiliations:** a Life Sciences Institute, Biosafety Level-3 Laboratory, Guangxi Medical University, Nanning, Guangxi 530021, China; b State Key Lab of Ultrasound in Medicine and Engineering, Chongqing Medical University, Chongqing 400016. China; c Department of Pulmonary and Critical Care Medicine, Institute of Respiratory Health, Frontiers Science Center for Disease-related Molecular Network, State Key Laboratory of Respiratory Health and Multimorbidity, West China Hospital, Chengdu, Sichuan 610041, China; d Department of Pathology, The First Affiliated Hospital of Guangxi Medical University, Nanning, Guangxi 530021, China; e Department of Respiratory and Critical Care Medicine, The First Affiliated Hospital of Chongqing Medical University, Chongqing 400016, China; f Chongqing Key Laboratory of Precision Medicine and Prevention of Major Respiratory Diseases, Chongqing 400037, China; g Core Facilities of West China Hospital, Sichuan University, Chengdu, Sichuan 610041, China; h Department of Systems Biology, Columbia University Medical Center, New York 10032, USA

Lung adenosquamous carcinoma (LASC) is distinct from lung adenocarcinoma (LUAD) and lung squamous cell carcinoma (LUSC), exhibiting higher malignancy and poorer prognosis. However, there is limited understanding of its single-cell heterogeneity, particularly in comparison to the single-cell heterogeneity of LUAD and LUSC. Here, we analyzed single-cell transcriptomic data from 34 tissue biopsy samples derived from 8 LUAD, 6 LASC, and 6 LUSC patients, and first present a single-cell resolution atlas for these distinct non-small cell lung cancer subtypes. We found that LUSC fibroblasts had higher heterogeneity compared with those from LUAD and LASC. Insulin-like growth factor binding protein 2-positive (*IGFBP2*^*+*^) fibroblasts exhibited the strongest interactions with macrophages, particularly a synergistic interaction with secreted phosphoprotein 1-positive (*SPP1*^*+*^) macrophages. Spatial relationship and crosstalk of these two subtypes were validated using independent datasets and *in vivo* experiments. Our findings offer a novel perspective on the biological mechanisms of tumor microenvironment in adeno-to-squamous transition, offering potential targets for therapeutic strategies in managing this disease progression.

LASC exhibits high heterogeneity, yet there has not been research to construct its single-cell atlas. The transdifferentiation from adenocarcinoma to squamous cell carcinoma involves a complex interplay of molecular mechanisms across multiple levels. Current research has predominantly focused on genomic characteristics, which alone do not fully elucidate the molecular mechanisms underlying adeno-squamous transdifferentiation.^1^ For instance, Arthur's discovery that LASC exhibits a LUAD-like genetic profile using whole-exome sequencing^2^ fails to account for the biological and clinical idiosyncrasies of LUAD different from LASC. Conversely, findings by Hongbin Ji et al that known LUAD markers gradually decrease, while LUSC markers increase in ranked LASC samples, provide insights into the gradual process of adeno-squamous transdifferentiation, supporting the lineage transition hypothesis that suggests pathological transformation within single tumors.^1^ Alvaro et al reported that LUSC transdifferentiation is primarily driven by transcriptional reprogramming rather than by mutational events,^3^ highlighting the paramount importance of transcriptional regulation in this transdifferentiation.

This study aimed to characterize the cellular heterogeneity and molecular events within the three major subtypes of non-small cell lung cancer. A total of 34 specimens, including 20 tumors and 14 matched adjacent normal tissues, were collected from 20 patients with pathologically diagnosed non-small cell lung cancer (8 LUAD, 6 LUSC, and 6 LASC). All patients were diagnosed with primary lung tumors and underwent no neoadjuvant therapy before surgery. The clinical stage of all patients was determined by the 8th TNM Classification. Clinical characteristics, including age, sex, smoking status, and pathological stage, are listed in Table S1.

The freshly resected tissues were processed to obtain single-cell suspensions, which were then subjected to single-cell RNA sequencing using the Chromium platform. The resulting data were mapped to the human reference genome and quality-controlled, followed by preprocessing with Seurat and Harmony. Copy number variations were inferred using the InferCNV (v1.17.0), and cell–cell communication was analyzed using CellChat (1.6.1). Pseudotime trajectory analysis was conducted with Monocle2 to map cell development. Finally, the prognostic value of certain genes was assessed using bulk transcriptomic data from TCGA and GEO databases, with survival and correlation analyses performed using GEPIA2 and R packages.

Our study presents a comprehensive single-cell transcriptome analysis of the tumor microenvironment across three subtypes of non-small cell lung cancer: LUAD, LUSC, and LASC. A total of 34 lung specimens were collected, resulting in the analysis of 123,234 cells post-quality control (Fig. 1A). The cells were categorized into 17 distinct groups (Fig. 1B; Fig. S1A), with a particular focus on fibroblasts due to their varying proportions across cancer subtypes (Fig. S1B). The study identified significant differences in the proportion of fibroblasts with single-cell copy number variants (Fig. 1C), with the highest levels observed in LUSC (Fig. S1C), suggesting a role in cancer progression. Pseudotime analysis revealed distinct branches for each non-small cell lung cancer subtype (Fig. 1D), with LASC fibroblasts at an intermediate stage, hinting at a transformational role. Additionally, 7354 differentially expressed genes were identified, with 635 potentially regulating fibroblast phenotype (Fig. S2A and S2B). Gene Ontology analysis pointed to enrichment in RNA/protein binding and RNA splicing, implicating RNA binding proteins and transcription factors in fibroblast function (Fig. S2C).

**Figure 1:**
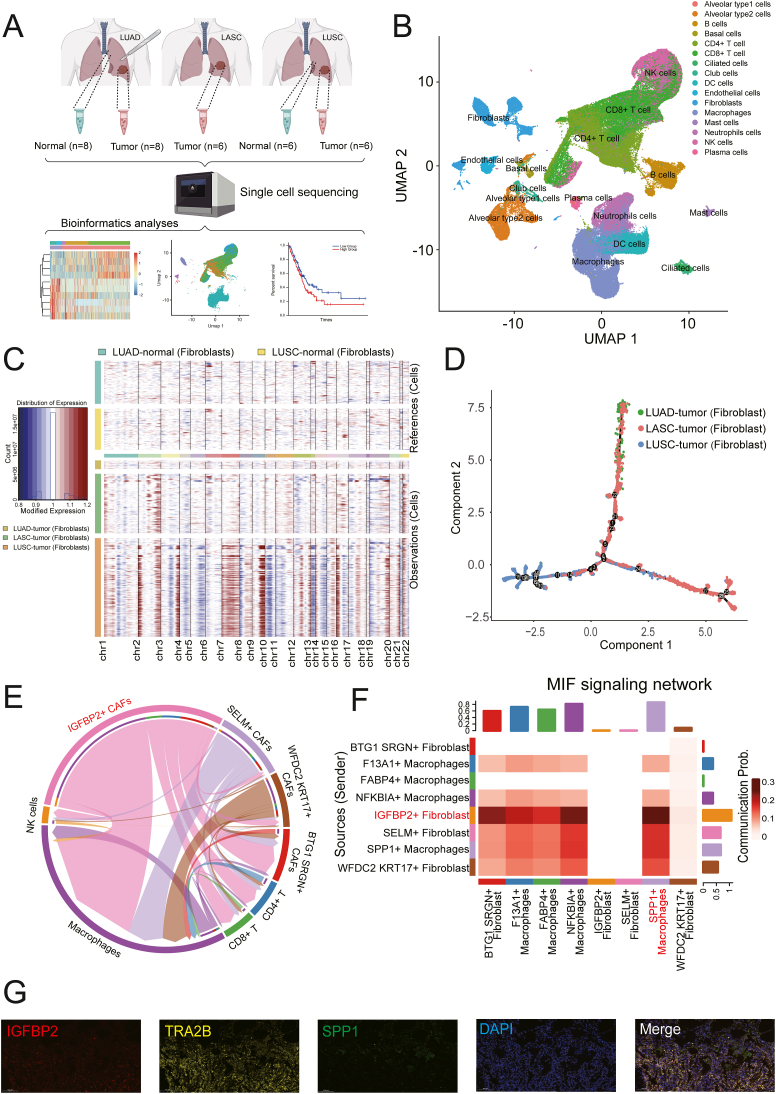
Roles of SPP1^+^ Mϕ in LASC. **(A)** Workflow of sampling, sequencing, and processes of bioinformatics analyses. **(B)** UMAP plot of the detailed cell types of all cells. **(C)** The overall SCNA patterns of the 6368 fibroblasts with SCNAs from tumors in 20 patients. Each row represents a single cell. **(D)** Developmental trajectory of fibroblasts, colored by group and cell subtype. **(E)** A chord diagram shows the communication between different cell subtypes. **(F)** Rows and columns of migration inhibitory factor (*MIF*) signaling network represent sources and targets of the cell communication. **(G)** Multicolor immunohistochemical staining for verifying the expression of antibody transcripts in different cell subtypes in LUAD. LASC, lung adenosquamous carcinoma; LUAD, lung adenocarcinoma; LUSC, lung squamous cell carcinoma; SCNA, somatic copy number alteration.

The study further classified fibroblasts into five distinct subtypes (Fig. S2D), revealing sample-specific clusters with unique characteristics. Communication analysis between fibroblasts and immune cells highlighted the *IGFBP2*^*+*^ fibroblasts' strong interaction with macrophages (Fig. 1E), suggesting a role in LUAD, LUSC, and LASC differentiation. The macrophage migration inhibitory factor (MIF) signaling pathway was identified as a key communicator (Fig. S2E). Functional annotation of differentially expressed genes between *SPP1*^*+*^ macrophages and *IGFBP2*^*+*^ fibroblasts revealed enrichment in pathways related to immune system signaling and extracellular matrix structure (Fig. S3A). Nine high-confidence genes were identified as potential regulators of RNA abnormalities (Fig. S3B), with elevated expression of secretory 61 beta (*SEC61B*), small nuclear ribonucleoprotein polypeptide E (*SNRPE*), and transformer 2 beta *TRA2B* correlating with poorer prognosis in patients with non-small cell lung cancer (Fig. S3C). Furthermore, the macrophages were divided into four different subtypes (Fig. S3D), and cell–cell communication analysis unveiled that *IGFBP2*^+^ cancer-associated fibroblasts demonstrated the most robust interactions with *SPP1*^+^ macrophages (Fig. 1F). Spatial analysis using immunofluorescent staining indicated a close spatial relationship between *IGFBP2*^*+*^ fibroblasts and *SPP1*^*+*^ macrophages in lung cancer tissues, with a significant correlation observed between *SPP1* and *IGFBP2* expressions in LUSC (Fig. 1G), suggesting their involvement in the transition between adenocarcinoma and squamous cell carcinoma (Fig. S3E).

Here, we find that the enrichment of *SPP1*-Mϕ in LUSC over LUAD is a gradual process, challenging the notion of an abrupt enrichment shift. Previous research suggests that the significant amplification of fibroblast growth factor receptor (*FGFR*) in squamous carcinoma, and the regulation of sex determining region Y-box 2 (*SOX2*)-induced differentiation by cancer-associated fibroblasts in LUSC,^4^ indicates that *IGFBP2*^+^ fibroblasts have the potential to regulate adeno-squamous transdifferentiation.^5^

In conclusion, our study offers new insights into understanding the role of fibroblasts and macrophages in the transdifferentiation from adenocarcinoma to squamous cell carcinoma, shedding light on the dynamic biological mechanisms involved. Future investigations are imperative to unravel the complexities underlying the mechanisms of adeno-squamous carcinoma transdifferentiation from multiple perspectives. Such a multifaceted analysis promises to shed light on the comprehensive cellular transformation mechanisms at play in the transition between adenocarcinoma and squamous cell carcinoma. This concerted effort will not only demystify the molecular intricacies of tumor transdifferentiation but also pave the way for innovative therapeutic strategies targeting the dynamic interactions within the tumor microenvironment.

## CRediT authorship contribution statement

**Yao Lin:** Writing – original draft, Formal analysis, Visualization, Data curation, Writing – review & editing, Methodology. **Yaxin Chen:** Visualization, Funding acquisition, Writing – review & editing, Resources, Conceptualization, Writing – original draft, Investigation. **Dandan Xiong:** Investigation, Writing – review & editing, Validation. **Jing Huang:** Validation, Writing – review & editing. **Hongmo Liu:** Writing – review & editing, Resources. **Yawen Qi:** Writing – review & editing, Resources. **Jinfeng Chen:** Validation, Writing – review & editing. **Jun Meng:** Visualization, Formal analysis, Writing – review & editing. **Yueqi Li:** Writing – review & editing, Formal analysis. **Jingyuan Yang:** Writing – review & editing, Validation. **Yi Bao:** Formal analysis, Writing – review & editing. **Wenxing Li:** Writing – review & editing, Methodology. **Li Yang:** Project administration, Writing – review & editing, Funding acquisition, Supervision, Conceptualization. **Sanqi An:** Supervision, Conceptualization, Writing – review & editing, Project administration, Writing – original draft, Methodology.

## Ethics declaration

This study protocol was approved by the Institutional Review Board of the First Affiliated Hospital of Guangxi Medical University (Ethics: 2024-S647-01), and all patients provided written informed consent.

## Data availability

All single-cell sequencing data for LUSC and LUAD are available via Science Data Bank (ScienceDB) by visiting https://doi.org/10.57760/sciencedb.02028. The generated LASC single-cell RNA-sequencing data of this study have been deposited in the Genome Sequence Archive (GSA) in BIG Data Center, Beijing Institute of Genomics (BIG) under accession number HRA007456. GSE31210, GSE8894, and GSE4716 for validation were available in GEO (https://www.ncbi.nlm.nih.gov/geo/) and GSA (https://ngdc.cncb.ac.cn/gsa/). The other resources used in this study are available from the corresponding authors upon reasonable request.

## Funding

This work was supported by the 10.13039/501100001809National Natural Science Foundation of China (No. 82203181 to L. Yang, 82300011 to Y. Chen) and the Chongqing Science and Technology Commission of China (No. CSTB2022TIAD-CUX0001 to L. Yang).

## Conflict of interests

The authors declared no competing interests.
