# Supplementary material for: Unveiling the roles of SPP1+ macrophage and IGFBP2+ fibroblast in lung adenosquamous carcinoma through single-cell analysis
Source: Genes Dis. 2025 Jul 24;13(1):101779. doi: 10.1016/j.gendis.2025.101779 (PMC12624626; doi:10.1016/j.gendis.2025.101779)
Supplement: Multimedia component 1 [file mmc1.pdf]

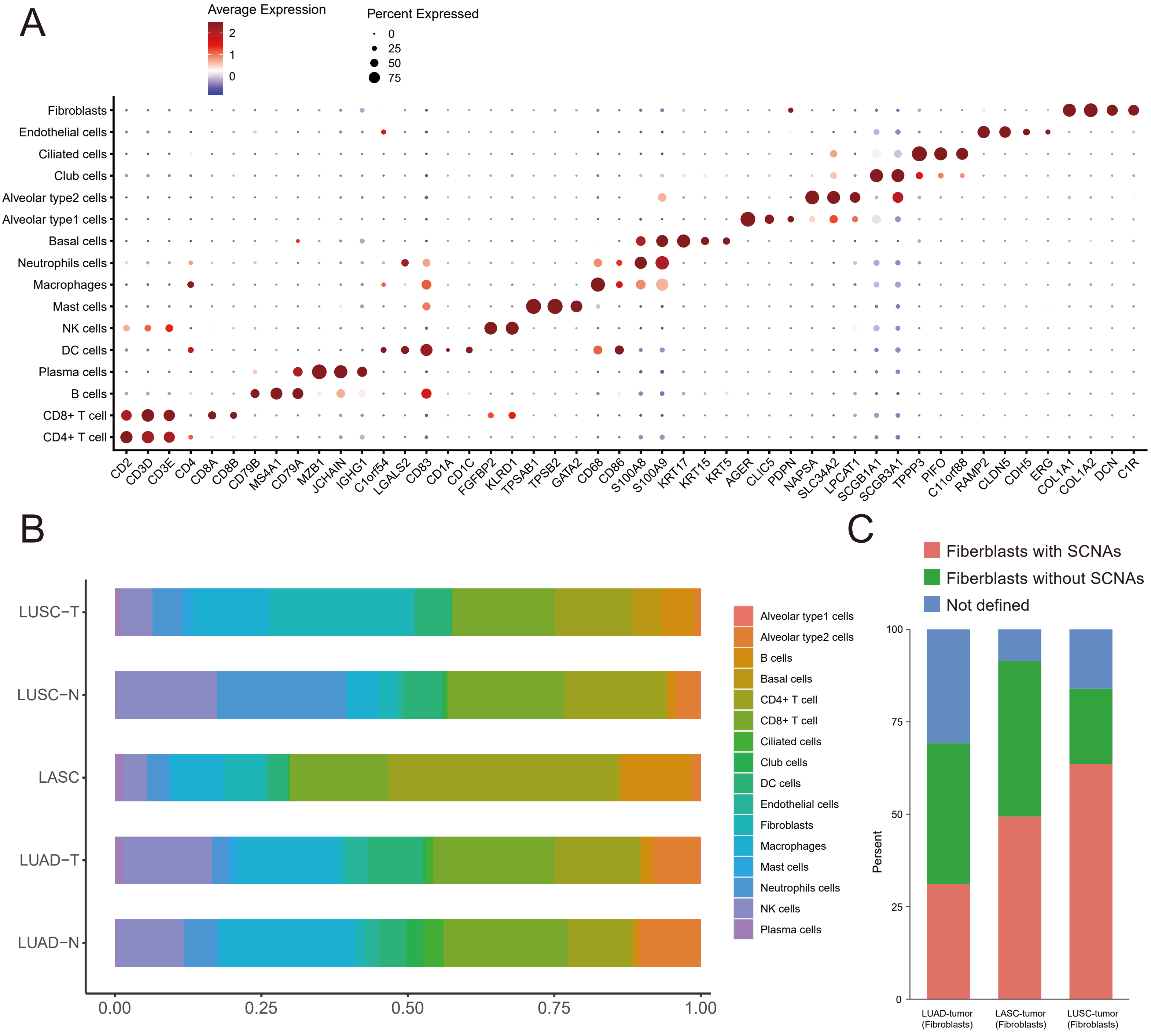

Figure S1 The frequency and features of all cells and fibroblasts cells with SCNAs in all cancer patients. A) Dot plot representing the expression of marker gene of the cell type. **B)** The proportion of cells that contributed by each sample to each cell type. **C)** The frequencies of fibroblasts with SCNAs among all fibroblasts. For all patients, Wilcoxon rank-sum test was used (p < 2.2×10-16).

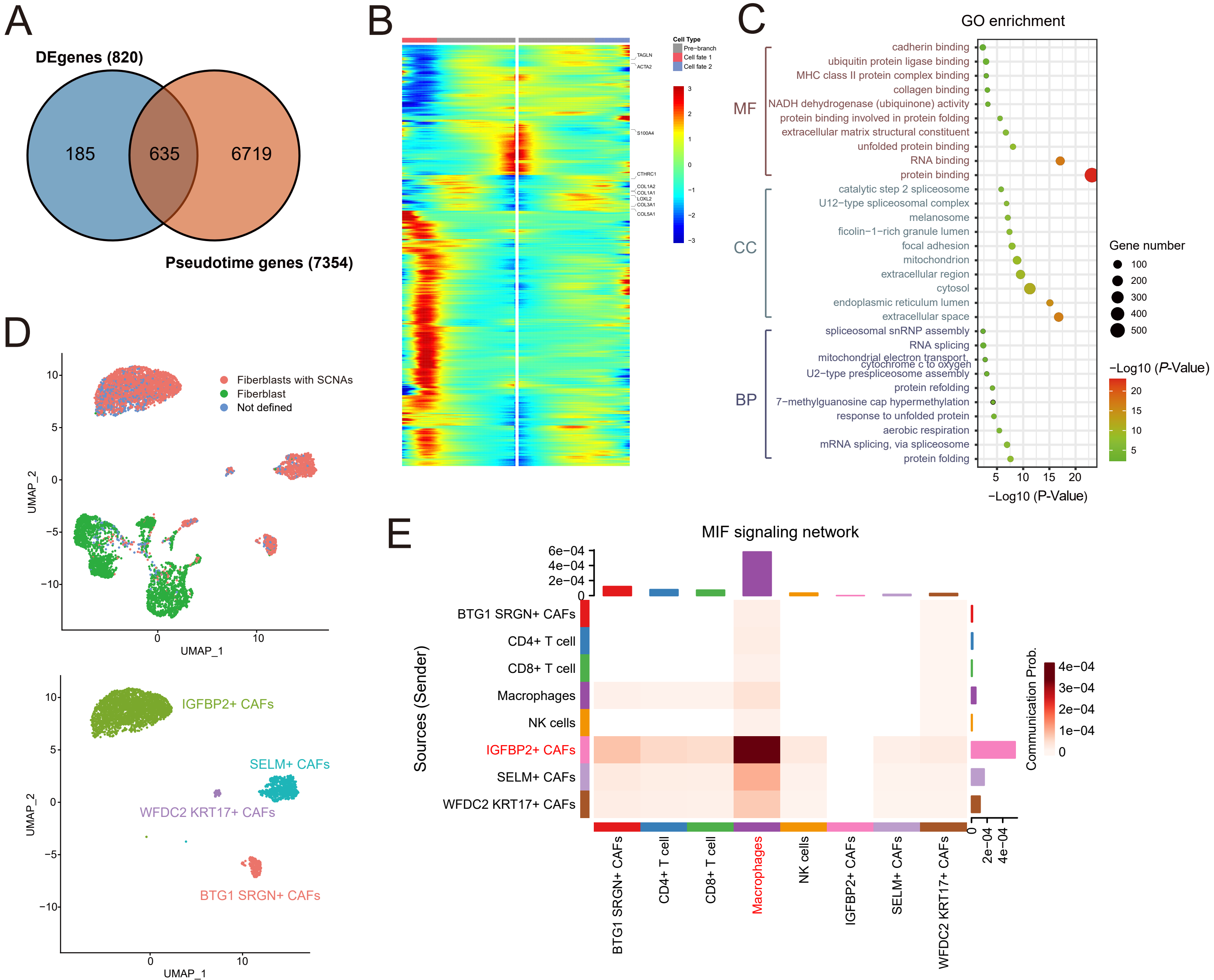

Figure S2 Subtype of fibroblasts in adeno-to-squamous transdifferentiation. A) Venn diagram demonstrating the identification of 635 DEGs. **B)** Heatmap of key 635 DEGs in different time. **C)** GO results of 635 DEGs top significantly enriched GO terms. **D)** UMAP plot of all fibroblasts which were colored by SCNA subtype and markers. **E)** Rows and columns of MIF signaling network represent sources and targets of the cell communication. Bar plots on the right and top represent the total interaction scores.

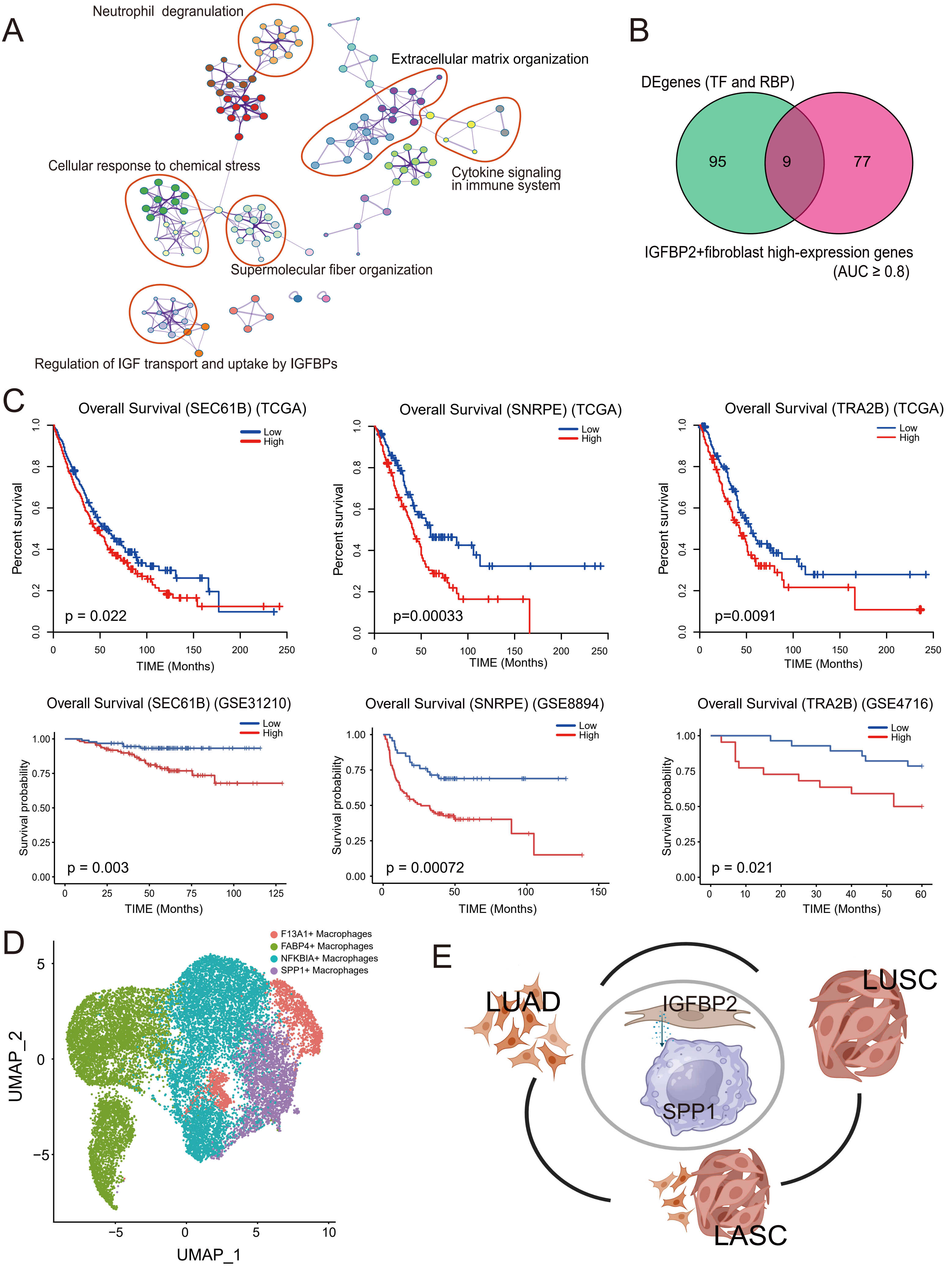

Figure S3 Potential regulators of fibroblasts in adeno-to-squamous transdifferentiation. A) Interaction network and functional annotation of DEGs. **B)** Venn diagram demonstrating the identification of nine potential regulators. **C)** Kaplan-Meier analysis showed worser overall survival in patients with tumors exhibiting high expression of *SEC61B*, *SNRPE* and *TRA2B*. **D)** UMAP plot of all Macrophages cells which were colored by cell subtypes. **E)** The overall schematic diagram of the theoretical mechanism.

Table S1 Clinical information of patients with LUAD, LUSC, and LASC, including age, sex, tumor size, tumor stage, past history, family history, smoking history, etc.

ID	Type	Sex	Age	Smoking	Quit smoking	Family history cancer	History past Tumor	Operation time	MaxDiameter tumor (cm)	LymphNode metastasis	Distant transfer	TNM
LUAD_1	LUAD	F	55	0	0	0	0	2018.5.10	1.2	N0	M0	IA2
LUAD_2	LUAD	F	55	0	0	0	0	2018.8.16	2.4	N0	M0	IB
LUAD_3	LUAD	F	54	0	0	Father esophageal cancer	0	2018.8.17	2.2	N0	M0	IB
LUAD_4	LUAD	F	39	15x2	0	Father gastric cancer	0	2018.11.14	0.5	N0	M0	IA1
LUAD_5	LUAD	F	39	15x2	0	Father gastric cancer	0	2018.11.14	0.5	N0	M0	IA1
LUAD_6	LUAD	F	64	0	0	0	0	2020.5.11	2	N0	M0	IA2
LUAD_7	LUAD	M	62	40x20	0	0	0	2019.8.5	2.5	N0	M0	IA3
LUAD_8	LUAD	F	58	0	0	Mother Rectal cancer	0	2018.11.21	1.3	N2	M0	IA2
LASC_1	LASC	M	72	20*20	NA	0	0	2020.4.15	7.2	N2	M0	IIIB
LASC_2	LASC	F	66	0	NA	0	0	2020.3.24	2.4	N0	M0	IB
LASC_3	LASC	F	73	0	NA	0	0	2020.8.27	3.2	N1	M0	IIB
LASC_4	LASC	M	66	33*50	NA	0	0	2020.1.13	4.2	N3	M0	IIIB
LASC_5	LASC	F	60	0	NA	0	0	2019.9.18	4	N0	M0	IB
LASC_6	LASC	F	NA	NA	0	NA	NA	NA	NA	NA	NA	NA
LUSC_1	LUSC	M	76	40x20	1	Father esophageal carcinoma	0	2018.9.12	2.1	N0	M0	IB
LUSC_2	LUSC	M	53	30x20	1	0	0	2018.8.14	3.2	N0	M0	IB
LUSC_3	LUSC	M	61	30x10	1	0	0	2018.8.29	10	N2	M0	IIIA
LUSC_4	LUSC	M	62	50x20	1	0	0	2019.1.7	4.6	N2	M0	IIIA
LUSC_5	LUSC	M	49	20x20	1	0	0	2018.11.28	3.6	N0	M0	IIB
LUSC_6	LUSC	M	43	0	0	0	0	2019.7.23	6	N0	M0	IIB
