# Supplementary material for: Unveiling the roles of SPP1+ macrophage and IGFBP2+ fibroblast in lung adenosquamous carcinoma through single-cell analysis
Source: Genes Dis. 2025 Jul 24;13(1):101779. doi: 10.1016/j.gendis.2025.101779 (PMC12624626; doi:10.1016/j.gendis.2025.101779)
Supplement: Multimedia component 2 [file mmc2.docx]

**Extended Materials**

**Sample preparation**

All freshly obtained resected tissues were immediately rinsed with Hank’s Balanced Salt Solution (HBSS, Life Technologies) and shredded on ice into smaller pieces with collagenase I/IV in HBSS. They were then incubated for 30 minutes at 37 °C with manual shaking per 10 minutes. The digested tissues were subsequently passed through a 70-μm nylon mesh filter to obtain cell suspensions, which were centrifuged at 500g for 5 minutes at 4 °C. Following supernatant removal, the pelted cells were suspended in red blood lysis buffer. The cells were then washed with HBSS and then resuspended in a buffer solution containing of 0.04% BSA in PBS. After dead cells removal through flow cytometry, the remaining cell suspensions were directly subjected to single-cell RNA-seq processing according to the manufacturer’s instructions. Alternatively, cell suspensions were frozen in 20% Dimethyl Sulfoxide (DMSO) and Fetal Bovine Serum (FBS), with cDNA library construction completed within 24 hours.

**scRNA-seq library preparation and sequencing**

Single-cell suspensions were processed into barcoded scRNA-seq libraries using the Chromium Single Cell 3’Library, Gel Bead & Multiplex Kit and Chip Kit (10x Genomics), according to the manufacturer’s instructions. Each sequencing-ready library, with approximately 5,000 cells, underwent purification with SPRIselect, quality control for size distribution, and yield (LabChip GX Perkin-Elmer) and quantification using quantitative PCR (KAPA). Libraries were sequenced on an Illumina NovaSeq-6000 platform, and each sample.

**scRNA-seq data analysis**

Single-cell RNA sequencing data were mapped to human reference genome (GRCh38) using CellRanger (v4.0.0) with default parameters. We then performed quality control on the single-cell data of all LUAD, LASC and LUSC samples, ensuring that cells met the following criteria: total counts less than 15,000, number of detected features between 500 and 4,000, mitochondrial gene ratio below 10%, and red blood cell gene ratio less than 1%. Subsequently, we utilized Seurat and Harmony ^1^ to preprocess the scRNA-seq data. This involved normalization, correction for batch effects, and clustering to facilitate downstream analyses. We found all those cells were divided into 17 distinct cellular groups, including alveolar type I cells (AT1, *N*=95), alveolar type II cells (AT2, *N*=5,605), B cells (B, *N*=5,023), basal cells (*N*=961), CD4+ memory T cells (*N*=16,396), CD4+ T cells (CD4, *N*=4,542), CD8+ naive T cells (*N*=20,227), CD8+ T cells (CD8, *N*=3,932), ciliated cells (Cilia, *N*=2,071), club cells (Club, *N*=10,548), endothelial cells (EC, *N*=2,153), fibroblasts (*N*=7,514), macrophages (*N*=18,055), mast cells (*N*=934), neutrophils cells (*N*=10,753), natural killer cells (NK, *N*=12,570), and plasma cells (*N*=1,855).

**CNV**

We inferred CNV using non-malignant cells (immune cells) as reference to estimate the mutations of malignant cells based on R package InferCNV (v1.17.0). To distinguish malignant and non-malignant cell types, we inferred CNV for each cell type based on the average expression of 100 genes in each chromosomal region using inferCNV. For fibroblasts, we used immune cells from three tumor samples as references.

**Cell–cell communication analysis**

The CellChat package [1.6.1] ^2^, a versatile toolkit for analyzing intercellular communication networks from scRNA-seq data, includes ligand-receptor interaction databases for human and mouse cells. It evaluates signaling inputs and outputs among cell clusters. Additionally, it incorporates a comprehensive signaling molecule interaction database, considering various structural compositions of receptor-ligand interactions. Through differential gene expression analysis and calculation of intercellular communication probabilities, CellChat facilitates the identification of statistically significant intercellular communications. We calculate and predict fibroblast clusters and associated immune cell clusters interactions ^3^. Then, we used netVisual_circle and netVisual_bubble functions to visualize the strength of cell-cell communication networks. All parameters are set to their default values.

**Pseudotime trajectory analysis**

The Monocle2 package ^4^ was employed for pseudotime analysis to map the differentiation trajectory of cell development. It introduced a pioneering approach of sequencing individual cells in pseudotime, leveraging the asynchronous processes of cells to align them along trajectories representing biological phenomena such as cell differentiation. Initially, the UMI matrix was extracted from the Seurat object, and then the newCellDataSet function was applied to generate the object, with the lowerDetectionLimit set as 0.1. We used DDRTree dimensionality reduction for cell sorting of fibroblasts in LUAD, LASC, and LUSC.

**Survival and correlation analysis**

We utilized bulk transcriptomic data from TCGA and GEO database to evaluate the prognostic value of interested genes in each NSCLC subtype. For patients in the TCGA-LUAD and TCGA-LUSC cohort, we directly conduct survival and correlation analysis in the database GEPIA2 (http://gepia2.cancer-pku.cn). For GEO datasets, we performed the overall Kaplan-Meier (KM) survival analysis by R package survival, which allows for the estimation of survival probabilities over time and the comparison of survival curves between different subtypes.

Refernces

1. Korsunsky I, Millard N, Fan J, et al. Fast, sensitive and accurate integration of single-cell data with Harmony. *Nat Methods.* 2019;16(12):1289-1296.

2. Jin S, Guerrero-Juarez CF, Zhang L, et al. Inference and analysis of cell-cell communication using CellChat. *Nature Communications.* 2021;12(1).

3. Mu G, Zhang W, Huang J, Chen Z, Wang J. Research Status of Tumor-associated Fibroblasts Regulating Immune Cells. *Zhongguo Fei Ai Za Zhi.* 2022;25(3):207-213.

4. Qiu X, Mao Q, Tang Y, et al. Reversed graph embedding resolves complex single-cell trajectories. *Nature Methods.* 2017;14(10):979-982.
